# Uracil–DNA Glycosylase from *Beta vulgaris*: Properties and Response to Abiotic Stress

**DOI:** 10.3390/ijms26178221

**Published:** 2025-08-24

**Authors:** Daria V. Petrova, Maria V. Zateeva, Lijun Zhang, Jiajia Zhang, Ying Zhao, Natalya V. Permyakova, Alla A. Zagorskaya, Vasily D. Zharkov, Anton V. Endutkin, Bing Yu, Chunquan Ma, Haiying Li, Dmitry O. Zharkov, Inga R. Grin

**Affiliations:** 1 Siberian Branch of the Russian Academy of Sciences Institute of Chemical Biology and Fundamental Medicine, 8 Lavrentieva Ave., 630090 Novosibirsk, Russia; dpetrova@niboch.nsc.ru (D.V.P.); zateeva2000@bk.ru (M.V.Z.); aend@niboch.nsc.ru (A.V.E.); 2 Engineering Research Center of Agricultural Microbiology Technology, Ministry of Education & Heilongjiang Provincial Key Laboratory of Plant Genetic Engineering and Biological Fermentation Engineering for Cold Region & Key Laboratory of Molecular Biology, College of Heilongjiang Province & School of Life Sciences, Heilongjiang University, Harbin 150080, China; zlj980821@163.com (L.Z.); zhangjiajia1664@163.com (J.Z.); 13339468678@163.com (Y.Z.); 2004078@hlju.edu.cn (B.Y.); chqm@hlju.edu.cn (C.M.); lihaiying@hlju.edu.cn (H.L.); 3 Department of Natural Sciences, Novosibirsk State University, 2 Pirogova St., 630090 Novosibirsk, Russia; 4 Siberian Branch of the Russian Academy of Sciences Institute of Cytology and Genetics, 10 Lavrentieva Ave., 630090 Novosibirsk, Russia; puh@bionet.nsc.ru (N.V.P.); zagorska@bionet.nsc.ru (A.A.Z.); 5 Biology Department, Tomsk State University, 36 Lenina Ave., 634050 Tomsk, Russia; arthropodae01@gmail.com

**Keywords:** DNA damage, DNA repair, DNA glycosylases, uracil–DNA glycosylase, plants, sugar beet, abiotic stress

## Abstract

Uracil−DNA glycosylases (UNGs) are DNA repair enzymes responsible for the removal of uracil, a canonical RNA nucleobase, from DNA, where it appears through cytosine deamination or incorporation from the cellular dUTP pool. While human and *Escherichia coli* UNGs have been extensively investigated, much less is known about their plant counterparts, of which UNGs from *Arabidopsis thaliana* are the only studied examples. Here, we show that in sugar beet (*Beta vulgaris* L.), an important crop species, cold and salt stress induce the expression of the *UNG* gene (*BvUNG*) and modulate the level of the uracil-excising activity in the roots. Purified recombinant BvUNG efficiently removes uracil from DNA both in vitro and in an *E. coli* reporter strain but does not excise 5-hydroxyuracil, 5,6-dihydrouracil, or 5-hydroxymethyluracil. The activity is abolished by Ugi, a protein UNG inhibitor from PBS1 bacteriophage, and by a mutation of a conserved active site His residue. Structural modeling shows the presence of a disordered N-tail prone to undergo phase separation, followed by a long α helix oriented differently from its counterpart in human UNG. Overall, BvUNG is a functional uracil–DNA glycosylase that might participate in the response to abiotic stress.

## 1. Introduction

**Figure 1:**
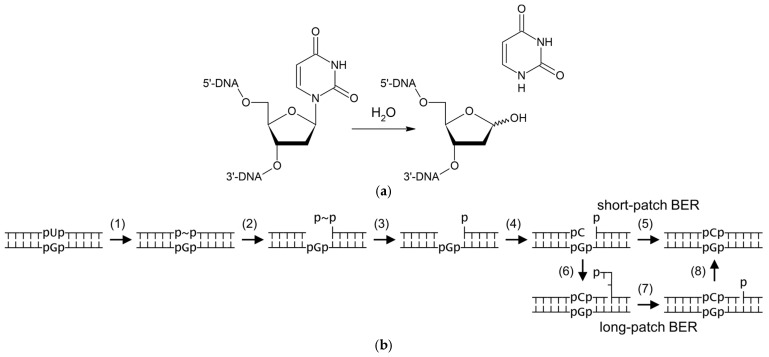
Reaction catalyzed by uracil–DNA glycosylases (**a**) and general scheme of uracil BER (**b**). Steps in (**b**) correspond to U base excision (1), AP endonuclease cleavage (2), dRP removal (3), normal dNMP incorporation (4), nick ligation (5 and 8), DNA synthesis with strand displacement (6), and flap excision (7). In (**b**), p is internucleoside phosphate (only those around the damaged base pair are shown for clarity), ~ stands for abasic deoxyribose.

Uracil–DNA *N*-glycosylases (UNGs) are DNA repair enzymes that catalyze the removal of uracil, a normal RNA component, from DNA where it arises through cytosine deamination or by incorporation from the cellular metabolic dUTP pool [1,2,3,4]. UNGs are present in all cellular life forms and even in some viruses [5]. As a DNA glycosylase, UNGs initiate the base excision repair (BER) pathway (see [6,7,8,9] for recent reviews of BER in different organisms) by hydrolyzing the *N*-glycosidic bond of 2′-deoxyuridine in DNA, forming an apurinic/apyrimidinic (AP) site (Figure 1a,b). The 5′-phosphodiester bond of the AP site is then cleaved by specialized AP endonucleases, leaving a nick flanked by a 3′-hydroxyl end and a 2′-deoxyribose-5′-phosphate (dRP) remnant. The latter is removed by a dRPase (either a separate enzyme or an activity present in repair DNA polymerases), and a normal dNMP is incorporated. The remaining nick can be ligated immediately (short-patch BER; Figure 1b), or, alternatively, the polymerase can insert several nucleotides, displacing a downstream flap, which is later trimmed by a flap endonuclease to leave a ligatable nick (long-patch BER; Figure 1b).

While uracil is steadily produced in DNA through spontaneous deamination and dUMP incorporation, other sources of this lesion are cellular stress-related. For example, reactive nitrogen species, such as nitric acid/nitrite or its anhydride N_2_O_3_, which easily convert C, A, and G bases to U, hypoxanthine, and xanthine, respectively, can be produced in the cell from the biological signaling molecule, nitric oxide, or by metabolic reduction in nitrate [10,11]. Another important source of U in DNA, at least in mammalian cells, is an improper activation of tightly regulated AID/APOBEC family cytidine deaminases, which normally participate in RNA editing, antiviral defense, and immunoglobulin gene somatic hypermutation [12,13].

Although UNG homologs from human (hUNG) and *Escherichia coli* (EcoUng; note that we use UNG and Ung capitalization for human and *E. coli* proteins, respectively, to keep in line with the nomenclature rules and the bulk of the literature) have been extensively studied, much less is known about UNGs from other major branches of life. In particular, plants experience a wide range of abiotic stresses, the relative importance of which is quite different from mammals and bacteria. Heavy metals, bright light, salt stress, and cold and heat stress trigger the production of reactive oxygen species and reactive nitrogen species (RNS) in plants, resulting in DNA damage [14,15,16]. Moreover, excessive use of nitrogen fertilizers, subject to nitrification by soil bacteria, also produces large amounts of RNS [17,18]. Thus, nucleobase deamination in DNA, in particular the C→U conversion, may be a common consequence of abiotic stress and nutrient imbalance in plants. *Arabidopsis thaliana* UNG (AtUNG), encoded by the At3g18630 gene, so far remains the only plant UNG cloned and characterized biochemically and genetically with respect to its specificity, reaction mechanism, and participation in the BER pathway [19,20,21,22,23]. *AtUNG* deficiency leads to mitochondrial genome instability [21]. At the same time, *AtUNG* knockout confers resistance to 5-fluorouracil, a thymidylate synthase inhibitor, likely by preventing excessive degradation of genomic DNA that accumulates high levels of uracil after such treatment [20,22,23]. However, less than twofold changes in *AtUNG* expression have been detected in transcriptomic studies after various kinds of DNA damage, temperature, and osmotic stress [24,25,26,27,28,29]. Thus, the role of UNGs in plant stress response remains unclear.

Sugar beet (*Beta vulgaris* L.) is an important food crop in cold and moderate climate zones worldwide. Cultivated sugar beet is often exposed to many kinds of abiotic stress, including low temperatures, drought, and excessive soil salinity. An apomictic *B. vulgaris* line, M14, which carries an extra copy of chromosome 9 from a close wild beet relative, *B. corolliflora* Zosimovic ex Buttler [30], was found to be significantly more resistant to these stress factors. Proteomic and transcriptomic studies revealed increased expression, either constitutive or abiotic stress-induced, of oxidative and glycation stress protection proteins, such as Cu,Zn-superoxide dismutase, thioredoxin peroxidase, and glyoxalase in M14 plants [31,32,33,34,35]. Earlier, in an ongoing effort to identify genes residing on chromosome 9 that could contribute to abiotic stress response, we had cloned *UNG* cDNA from *B. vulgaris* M14 [36]. Here, we report characterization of uracil–DNA glycosylase activity in *B. vulgaris* plants and the properties of the recombinant *B. vulgaris* M14 UNG (BvM14UNG).

## 2. Results

### 2.1. Uracil–DNA Glycosylase Activity in B. vulgaris

In addition to the model plant *A. thaliana*, many monocot and dicot flowering plants including rape (*Brassica napus* L.), turnip (*B. rapa* L.), carrot (*Daucus carota* L.), potato (*Solanum tuberosum* L.), tobacco (*Nicotiana tabacum* L.), garden pea (*Pisum sativum* L.), thyme (*Thymus vulgaris* L.), African violet (*Saintpaulia ionantha* H.Wendl.), onion (*Allium cepa* L.), wheat (*Triticum aestivum* L.), and maize (*Zea mays* L.) were reported to possess robust uracil–DNA glycosylase activity in the extracts from different organs and subcellular organelles [37,38,39,40,41,42,43,44]. However, to our knowledge, no such data is available for any species from the order Caryophyllales, which, in addition to sugar beet, includes such valuable food cultures as spinach, quinoa, buckwheat, rhubarb, and sorrel. Thus, we have assayed for the presence of a uracil-excising activity in total extracts of leaves and roots of young (19 days after planting) *B. vulgaris* plants. As a substrate in the reactions with crude extracts, we used a single-stranded 23-mer oligonucleotide that carried a fluorescent 5(6)-carboxyfluorescein moiety at the 5′-end and had two 3′-terminal internucleoside phosphodiester bonds replaced with phosphorothioate linkages to minimize non-specific degradation by cellular nucleases (23Ups, Table 1). AtUNG, hUNG, and EcoUng efficiently process single-stranded DNA substrates [2,3,4]. Since UNGs do not cleave the DNA backbone, the reaction products were treated with hot alkali to induce β-elimination at the AP sites formed after the uracil removal.

**Figure 2:**
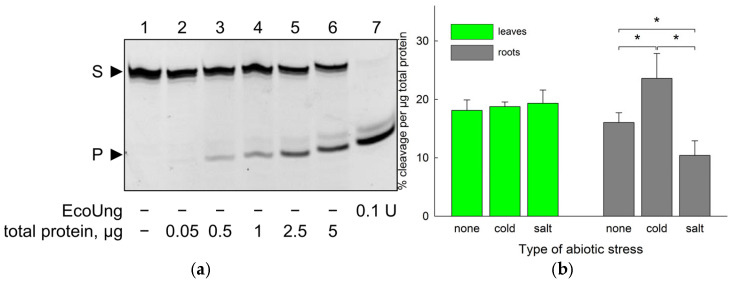

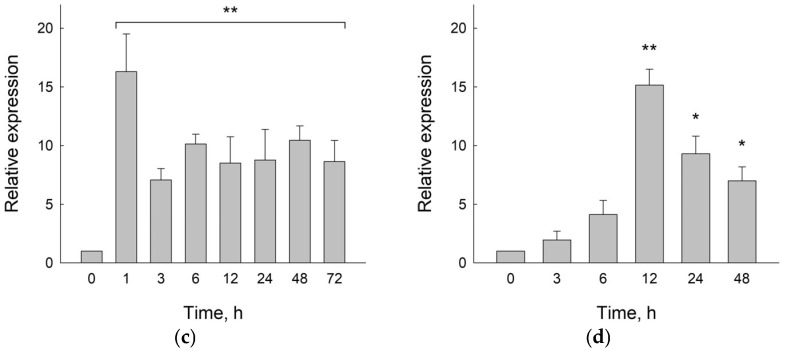
Uracil–DNA glycosylase activity and expression in *B. vulgaris* extracts. (**a**) Representative gel showing the cleavage of the 23Ups oligonucleotide substrate by various amounts of *B. vulgaris* root extract. S, 23-mer substrate; P, 10-mer product. (**b**) Specific uracil excision activity in *B. vulgaris* leaves and root extracts after cold and salt stress. Mean ± s.d. is shown (*n* = 9). *, *p* < 0.05 (Student’s *t*-test). (**c**) Expression level of *BvM14UNG* in the seedlings after cold stress, normalized for 0 h treatment. Mean ± s.d. is shown (*n* = 3). **, *p* < 0.01 vs. 0 h (Student’s *t*-test). (**d**) Expression level of *BvM14UNG* in the seedlings after salt stress, normalized for 0 h treatment. Mean ± s.d. is shown (*n* = 3). *, *p* < 0.05, **, *p* < 0.01 vs. 0 h (Student’s *t*-test).

As can be seen from Figure 2a, treatment of the uracil-containing oligonucleotide substrate with *B. vulgaris* extracts resulted in the production of a shorter species in a concentration-dependent manner, which co-migrated with the product of cleavage of the same substrate by EcoUng. The activity was present in both leaves and roots, and the specific activity in these organs was similar in the absence of abiotic stress (Figure 2b).

When the plants were exposed to cold stress (5 h at 4 °C) or salt stress (300 mM NaCl for 12 h), the response in the leaves and the roots was different. The relative uracil–DNA glycosylase activity did not change in the leaves after either treatment (Figure 2b). In contrast, the activity in the roots was increased by the cold stress and decreased by the salt stress (Figure 2b), although the magnitude of the effect was rather moderate (~1.5-fold change in both cases).

To complement the enzyme activity data, we have followed the time course of *BvM14UNG* mRNA levels in the whole plant upon abiotic stress. The plants were exposed to cold stress for 1 h, 3 h, 6 h, 12 h, 24 h, 48 h, or 72 h and to salt stress for 3 h, 6 h, 12 h, 24 h, or 48 h (200 mM NaCl was used in these experiments to enable longer observations without plant deterioration). During the cold stress, the expression of *BvM14UNG* sharply peaked at 1 h and then decreased but remained 7–10-fold higher than in the unexposed plants until the end (Figure 2c). The salt-stressed plants showed a more gradual rise followed by a decrease, with the expression of *BvM14UNG* peaking at 12 h and remaining ~7-fold above the unexposed control by 48 h (Figure 2d).

Thus, *B. vulgaris* possesses functional uracil–DNA glycosylase, which responds to abiotic stresses at the expression and enzyme activity levels. However, these responses hardly correlate with each other. Whereas salt and cold stress induce BvUNG at the mRNA level, induction of the enzyme activity is limited to cold stress in roots, whereas salt stress in roots actually suppresses the activity.

### 2.2. Predicted Structure of BvUNG

**Figure 3:**
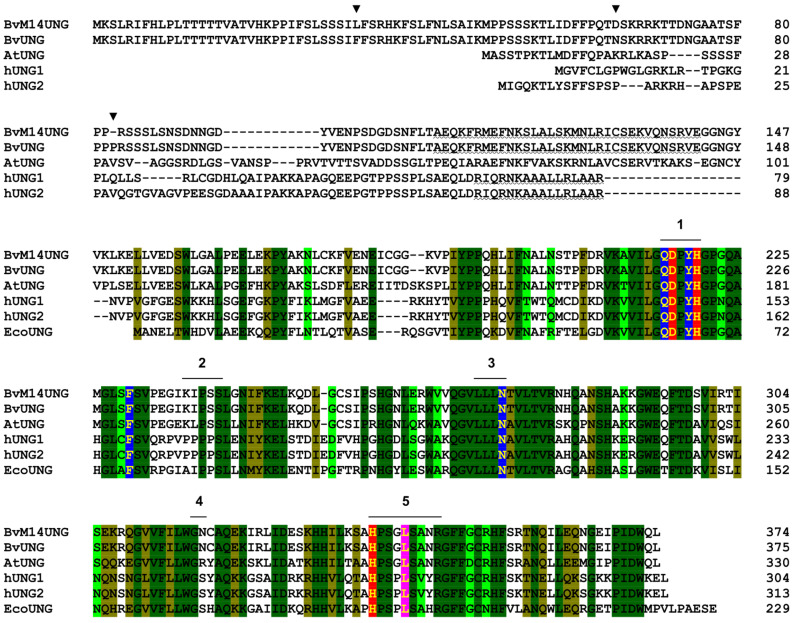
Alignment of UNGs from *B. vulgaris*, *A. thaliana*, human, and *E. coli*. Conservative positions across all sequences are shown in a light green (less conserved) to dark green (most conserved) gradient. Numbers indicate the water-activating loop (1), the proline-rich loop (2), the uracil recognition loop (3), the Gly-Ser loop (4), and the minor groove intercalation loop (5) as defined for hUNG in [50,51]. Residues forming the uracil recognition pocket are highlighted in blue, catalytic residues are in red, and helix-intruding residues are in magenta. Arrowheads mark the different positions between BvM14UNG and BvUNG. The wavy underlines show the α_0_ helix generated by AlphaFold (BvUNG, this work) or observed by NMR (hUNG [52]).

In *A. thaliana*, two proteins were shown to have uracil–DNA glycosylase activity, namely, AtUNG and AtMBD4L, with the former regarded as the main uracil repair enzyme [20,21,22,45,46,47]. Encouraged by the detection of the activity in *B. vulgaris* plants, we sought to characterize UNGs from this species in more detail. A comparison of the predicted protein sequences of BvM14UNG [36] and that of the reference *B. vulgaris* genome assembly EL10.2 [48] (NCBI reference sequence XP_048492575.1) reveals three changes in the poorly conserved N-terminal tail of the protein (Figure 3). However, the same variations encompassing these positions can be found in other *B. vulgaris* genome assemblies (N65D and delP83 in cultivar KWS2320 [49]; F33L in cultivar PI 607898, unpublished, BioProject PRJNA1010308), so we assume that they are part of the natural species variability and that the reference sequence XP_048492575.1 can be used meaningfully to analyze the structure and properties of *B. vulgaris* UNG. For the sake of brevity, we will further refer to this protein as BvUNG independently of the strain/cultivar of origin. In a phylogenetic analysis of plant UNGs (Supplementary Figure S1, Supplementary Files S1 and S2), BvUNG expectedly clustered with the homologs from *Chenopodium quinoa* and *Spinacia oleracea* (both are members of the order Caryophyllales, together with *B. vulgaris*). Among all dicots, this branch was the closest to UNGs from monocots and non-angiosperms (Supplementary Figure S1).

**Figure 4:**
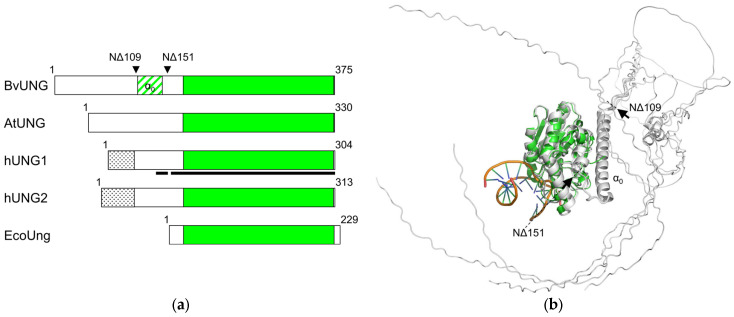

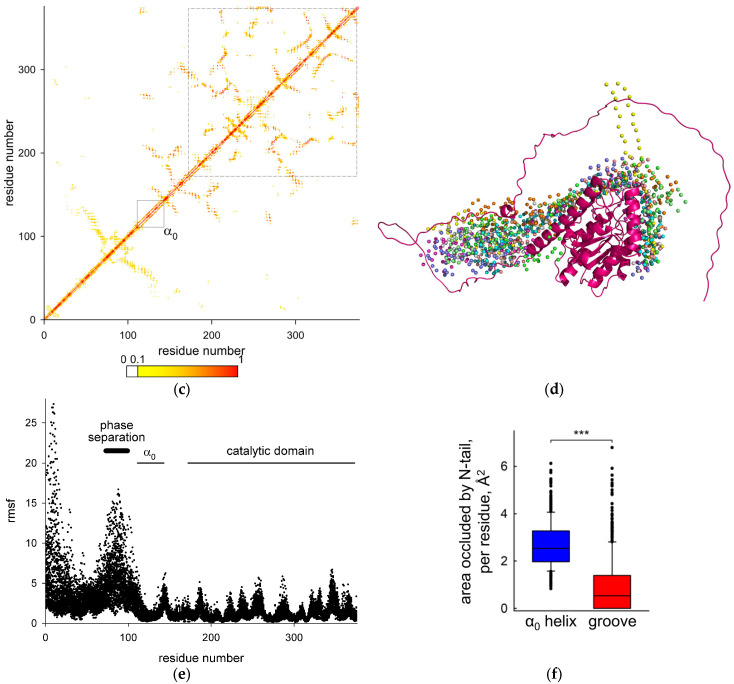
Structure of BvUNG. (**a**) Scheme of domain organization of BvUNG, AtUNG, hUNG1, hUNG2, and EcoUng proteins. The green box is the UDG-F1-like catalytic domain (cd10027 in the Conserved Domain Database [55]). The hatched green box is the α_0_ helix in the N-terminal tail of BvUNG predicted by AlphaFold. Arrowheads mark the sites of truncation of BvUNG for overexpression in *E. coli*. In hUNG1 and hUNG2, the different N-terminal regions between the isoforms are shown as dotted boxes. The black bar under hUNG1 indicates the regions of human UNGs with the structure solved by crystallography or NMR [50,52]. (**b**) Overlay of the structure of hUNG bound to DNA (PDB ID 1SSP [50], green) and five AlphaFold-generated BvUNG models (gray). Arrows mark the sites of truncation of BvUNG. (**c**) Contact map averaged over all CABS-flex simulations. Hatched box, BvUNG catalytic domain; solid box, α_0_ helix. The colored bar shows the frequency of contacts. (**d**) An example of a population of conformations of the N-tail obtained in one simulation. The initial AlphaFold structure is shown as a cartoon representation. The Cα atoms (residues 1–110) from ten medioid structures of the most populated clusters are shown as colored balls, each color corresponding to one structure. The backbone traces are omitted for clarity. (**e**) RMSF plot of all CABS-flex simulations; each dot represents the RMSF of a single simulation sample consisting of 1000 structures. (**f**) Area of the α_0_ helix and the DNA-binding groove occluded by the N-tail of BvUNG in 500 medioid structures. ***, *p* < 0.001 (Mann–Whitney *U* test).

The human *UNG* gene produces two mRNA and protein isoforms due to alternative transcription initiation (Figure 4a). The shorter isoform, hUNG1, carries a canonical mitochondrial targeting sequence, and hUNG2 is classified as a nuclear isoform [53,54]. EcoUng is ~25% shorter and essentially consists of the catalytic domain only (Figure 3 and Figure 4a; here, we define the catalytic domain as the cd10027 UDG-F1-like domain in the Conserved Domain Database [55]). Only single mRNA and protein isoforms of AtUNG and BvUNG are known, and their N-terminal parts lack homology with either human isoform (Figure 3 and Figure 4a). At the same time, the catalytic domains of plant UNGs are highly similar to their human and *E. coli* counterparts, keeping intact the key catalytic residues and motifs 1–5, which recognize the Ura base, pinch the phosphodiester backbone to kink DNA and flip the damaged base into the enzyme’s active site, and fill the space left in the duplex after the base eversion (Figure 3). To assess the similarity of the three-dimensional structure of BvUNG to other members of the uracil–DNA glycosylase family, we have generated structure models of BvUNG using AlphaFold2 [56]. When superimposed over the structure of hUNG bound to DNA after uracil excision (PDB ID 1SSP [50]), the top five BvUNG models showed excellent agreement in their catalytic core structure (r.m.s.d. 1.220–1.376 Å over all heavy atoms) (Figure 4b). The DNA-binding interface was strongly conserved between BvUNG and hUNG. However, the N-terminal part (residues 1–154) remained mostly unfolded in the models, likely because of low sequence conservation and the lack of experimentally resolved structures. An exception was a stretch of residues 111–143, consistently folding into a long α-helix, which we have termed α_0_ (Figure 4b). Of note, the corresponding (but shorter) peptide from hUNG was studied by NMR in a complex with replication protein A p32 subunit and was also shown to form an α-helix [52]. To cross-check the AlphaFold predictions, we have generated another model of BvUNG using an algorithmically independent protein folding software, ESMFold v1.0.3 [57]. The model demonstrated excellent convergence with the AlphaFold models over the ordered part of the protein (all-atom RMSD 0.36–0.40 Å for the catalytic core), including the appearance and the position of the α_0_ helix (Supplementary Figure S2). We have also assured the robust emergence of the α_0_ helix performing ab initio folding of the isolated N-terminal part of BvUNG (residues 1–151) by all-atom Monte Carlo simulations in QUARK [58] (Supplementary Figure S3). Finally, the α_0_ helix emerged in the AlphaFold models of AtUNG and five other plant UNGs representing the main phylogenetic clusters (Supplementary Figure S4).

To obtain better insight into possible conformations of the disordered N-tail, we used CABS-flex 2.0, a coarse-grained Monte Carlo modeling tool [59,60]. For each of the five AlphaFold-generated models, we performed ten simulations, collecting a total of 50,000 structures. In the vast majority of low-energy models, the α_0_ helix was stable, and the N-tail folded into hairpin-like structures, compacting the occupied space (Figure 4c,d). Although the N-tail was more mobile than the catalytic domain and assumed a large range of conformations (Figure 4d,e), it was mostly located at the side of the catalytic core opposite to the DNA-binding surface of UNGs, leaving the protein–DNA interface minimally perturbed (Figure 4f). We also analyzed the BvUNG structure using ParSe v2, a neural network trained on an extensive set of intrinsically disordered proteins [61], to assess the propensity of the N-tail to induce liquid–liquid phase separation. Indeed, residues 73–99 were predicted by all ParSe classifiers as intrinsically disordered and prone to undergo phase separation (Figure 4e).

Many aspects of hUNG biology, including its activity, interactions with the replication machinery, and proteasomal degradation, are regulated by post-translational modifications, mostly Ser/Thr phosphorylation and Lys acetylation of the N-tail [62,63,64,65,66,67,68]. A phosphoproteome study of AtUNG [69] revealed phosphorylation at Ser46, which, however, is conserved neither in BvUNG nor hUNG (Figure 2). To obtain a better understanding of the possible regulation of BvUNG, we have used PTMGPT2, a large language model-based predictor of post-translational modification sites [70]. The predicted modifications and their positions are listed in Table 2; no other modifications (Tyr phosphorylation, Lys or Arg methylation, ubiquitination, Lys or Pro hydroxylation, *S*-palmitoylation, Val amidation) were suggested. Probable Ser/Thr modification sites were exclusively located in the N-tail, approximately in the same region where Ser46 is found in AtUNG. Moreover, several Lys residues in the α_0_ helix were predicted as possible sites of acetylation or other types of acylation. It can be inferred that the N-tail and the α_0_ helix are likely involved in the regulation of BvUNG functions through post-translational modification.

### 2.3. Activity of Recombinant BvUNG

**Figure 5:**
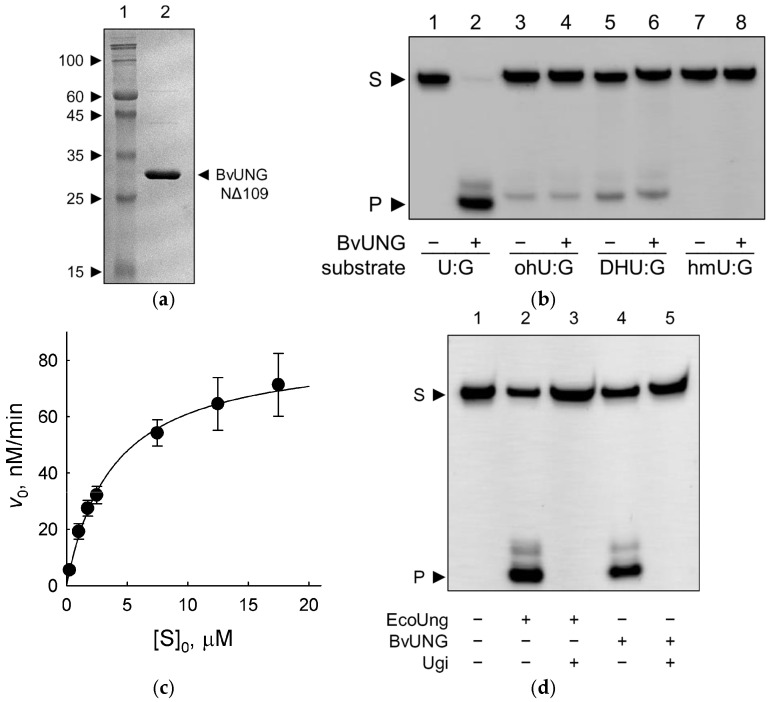
Activity of the recombinant BvUNG. (**a**) Purified BvUNG NΔ109 after 12% SDS-PAGE. The gel was stained with Coomassie Blue. Arrows show the molecular weights of the mobility markers. The expected molecular weight of BvUNG NΔ109 with a His-tag is 32 kDa. (**b**) Activity of BvUNG on DNA duplexes containing various damaged pyrimidine bases. (**c**) Representative plot of reaction velocity vs. substrate concentration for cleavage of the U:G substrate by BvUNG. (**d**) Inhibition of BvUNG by Ugi. S, substrate; P, cleavage product.

For a more thorough biochemical characterization of the activity of BvUNG, we have produced the recombinant protein expressed in *E. coli*. Since the disordered tails present in eukaryotic proteins often cause low expression or protein aggregation in bacterial cells, we constructed plasmids carrying inserts that encode full-length BvUNG and two N-terminally truncated variants, NΔ109 and NΔ151. In BvUNG NΔ109, the protein is clipped before the start of the α_0_ helix, so only the disordered part is missing (Figure 4a,b). In BvUNG NΔ151, the α_0_ helix is also removed, and the remaining part corresponds to the extensively studied truncated form of hUNG lacking isoform-specific parts [50,71,72,73,74,75,76,77,78,79,80,81,82]. Full-length BvUNG and BvUNG NΔ151 were produced as insoluble aggregates but we were able to purify soluble BvUNG NΔ109 (Figure 5a).

The activity of BvUNG NΔ109 was characterized on three oligonucleotide substrates: a single-stranded 23-mer carrying U in the 11th position and two duplexes forming either a U:A or a U:G pair (Figure 5b,c, Table 1 and Table 3). BvUNG efficiently converted U-containing substrates to AP sites, which could be revealed by DNA backbone cleavage after heating in the presence of 0.1 M NaOH. No cleavage at T residues was observed. Additionally, we have tested the activity on substrates containing oxidized U or T bases (5-hydroxyuracil, 5,6-dihydrouracil, and 5-hydroxymethyluracil) but did not see any cleavage above the background substrate decomposition upon hot alkali treatment (Figure 5b). Based on the specificity constant (*k*_sp_ = *k*_cat_/*K*_M_) measured under steady-state conditions, the U:G mispair was the best substrate due to its lower *K*_M_ value, whereas U:A and single-stranded U substrates were processed with a similar efficiency. The same preference for U:G over U:A double-stranded substrates has been reported for the human and *A. thaliana* UNG, although the human enzyme apparently excises U from single-stranded DNA even better than from a duplex [20,50,83].

Finally, we have inquired whether BvUNG is sensitive to bacteriophage PBS1 Ugi, a small (9.5-kDa) “DNA-mimic” protein [84] that tightly binds and specifically inhibits uracil–DNA glycosylases. U replaces T in the genome of PBS phages [85,86], so Ugi helps to prevent the degradation of the invading phage DNA. Ugi inactivates UNG family glycosylases but not their structural relatives, such as human TDG and SMUG1 or *E. coli* Mug enzymes, even though they also are capable of recognizing and removing U from DNA. As can be seen from Figure 5d, Ugi efficiently inhibited both EcoUng and BvUNG, confirming that the latter is a typical uracil–DNA glycosylase.

### 2.4. BvUNG Is Functional in an E. coli Reporter Strain

**Figure 6:**
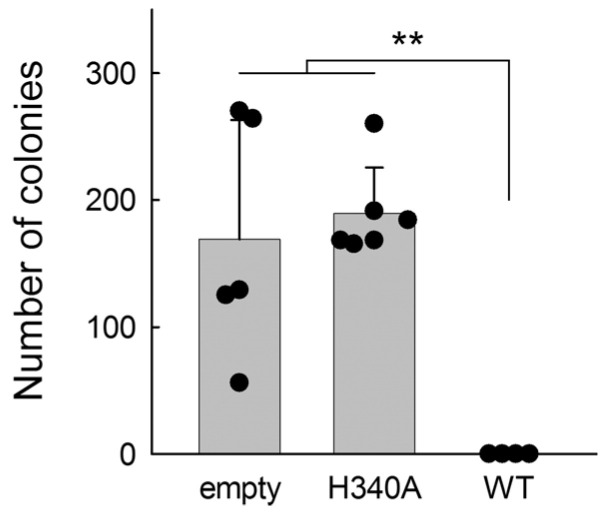
Survival of *E. coli* CJ236 transformed with the empty pET-15b plasmid or pET-15b carrying wild-type or H340A BvUNG insert. Mean ± s.d. (*n* = 4–6); **, *p* < 0.01 (one-way ANOVA with Holm–Bonferroni correction).

To complement our in vitro studies and test the activity of BvUNG in the intracellular environment, we have expressed full-length BvUNG in the *E. coli* CJ236 strain, which carries inactivating mutations in the *ung* and *dut* genes. Dut is a dUTP nucleotide hydrolase, normally engaged in the pyrimidine nucleotide biosynthesis pathway to convert dUTP to dUMP, the metabolic precursor of dTMP [87,88]. In the absence of both Ung and Dut, high levels of uracil accumulate in the genomic DNA, which is only moderately harmful unless active uracil–DNA glycosylase is introduced into these cells, causing lethal massive genome breakage [89,90]. Thus, we have transformed CJ236 cells with equimolar amounts of an empty pET-15b plasmid or pET-15b carrying an insert of full-length BvUNG, either wild-type or containing a H340A mutation, which removes the catalytic His340 residue. In EcoUng, the equivalent H187A mutation decreases the activity 150–300-fold [91], and in hUNG, a substitution of Leu for the corresponding His268 decreases the activity > 300-fold [92]. Of note, many pET series plasmids usually show leaky transcription of the inserts when grown on rich media, even in the absence of T7 RNA polymerase, allowing functional assessment of the encoded proteins in *E. coli* cells [93,94,95]. No significant difference in the CJ236 cells’ survival was observed for the empty plasmid and BvUNG H340A, while the cells transformed with wild-type BvUNG did not grow at all (Figure 6). Thus, full-length BvUNG is an active uracil–DNA glycosylase when expressed in *E. coli* cells.

## 3. Discussion

Uracil–DNA glycosylases represent an important line of defense against spontaneous and induced DNA damage. *E. coli ung* mutants show an elevated frequency of C→T transitions [96] and a moderately enhanced susceptibility to several antibiotics such as chloramphenicol, tetracycline, carbenicillin, and ceftazidime [97]. Baker’s yeast *ung1* knockouts or null mutants also predominantly accumulate C→T transitions [98], are hypersensitive to deamination-inducing agents (NaHSO_3_, NaNO_2_) [99] and to heat shock [100], and demonstrate changes in the amino acid metabolome [101]. In mammals, deficiency in UNGs manifests primarily in immune disorders since targeted C→U deamination and processing by UNGs is critical for somatic hypermutation and class switch recombination in immunoglobulin genes [4,102,103,104]. *Ung* knockout mice additionally display high sensitivity to folate deficiency, likely because of reduced dUMP→dTMP conversion and increased dUMP incorporation into DNA [105,106].

In comparison, the properties and functions of UNGs in plants have been studied rather scantily so far. The only species from which the gene was cloned and the recombinant protein purified and characterized is *A. thaliana* [20]. As an enzyme, AtUNG shows properties fairly similar to EcoUng and hUNG. It removes uracil from both single-stranded and double-stranded substrates, irrespective of the sequence and opposite-base context, does not excise damaged bases with bulky C5 substituents, and is inhibited by Ugi [20]. *AtUNG* T-DNA insertion mutants show increased resistance to 5-fluorouracil, a thymidylate synthase inhibitor that causes excessive accumulation of U in genomic DNA and chromosome fragmentation in UNG-positive cells [20]. Other than that, no overt phenotypes of *AtUNG* knockouts have been reported. Strikingly, neither the nuclear nor the mitochondrial genome of UNG-deficient *A. thaliana* accumulates point mutations, but mitochondrial DNA demonstrates increased recombination, suggesting that recombination contributes to uracil repair in plants, or at least in this species [21]. Inspection of the Gene Expression Omnibus transcriptomic database reveals that *AtUNG* expression responds to many biotic and abiotic factors such as fungal infection [107], respiratory stress [108,109], γ-irradiation [29], phosphate depletion [110], cold [25], salt and osmotic shock [111,112], regulatory signals including ethylene [113], and light [114,115,116,117,118,119]. However, the magnitude of these effects rarely exceeds twofold. In *A. thaliana* and in rice, *UNGs* are strongly downregulated during gamete development [120,121].

BvUNG provides the second example of plant UNGs characterized as a recombinant protein, and it would be instructive to compare its properties with its homolog from *A. thaliana*. Of note, we have produced an N-terminally truncated BvUNG, whereas AtUNG studied in [20] was a full-length protein carrying a His-tag and a short plasmid-encoded linker at its N-terminus. Nevertheless, the enzymatic properties of both proteins turned out to be similar. Both AtUNG and BvUNG are monofunctional DNA glycosylases with no AP lyase activity, appear to prefer U:G over U:A substrates and process U in single-stranded DNA, and are sensitive to Ugi. Neither BvUNG nor AtUNG removed modified pyrimidine bases other than U. In particular, both plant enzymes do not excise 5-hydroxyuracil, which is a minor substrate for hUNG and EcoUng [122,123]. Replacement of key catalytic residues (His340 in BvUNG, Asp173 in AtUNG, equivalent to Asp218 in BvUNG) eliminates the activity, supporting the reaction chemistry inferred from studies of hUNG and EcoUng, in which the His residue promotes uracil base leaving while Asp activates the catalytic water attacking C1′ from the opposite side.

The vast majority of biochemical studies on hUNG were performed with the truncated form of the protein, common for hUNG1 and hUNG2, which misses the N-tail (the first 84 residues in hUNG1 or 93 residues in hUNG2). This truncated form has full catalytic activity and is easily purified from overproducing bacterial cells [124]. Thus, it was surprising that the equivalent truncated variant of BvUNG was insoluble upon expression in *E. coli* and required the additional α_0_ helix for stability. Interestingly, the α_0_ helix is also predicted by AlphaFold in both AtUNG and hUNGs but is oriented differently in the human and the plant proteins. In BvUNG and AtUNG, it lies parallel to the surface of the catalytic domain, making extensive contacts with it, whereas in hUNG1/2, the helix is shorter and projects to the solution almost orthogonal to the protein globule. The reason for this difference seems to be the long insertion in the plant UNGs (residues 132–150 in BvUNG, see Figure 2), which allows the helix to fold back on the protein’s surface. Since hUNG2 interacts with replication protein A through the α_0_ helix [52,125,126], this may possibly reflect different protein–protein interactions for the human and plant enzymes.

At the organismal level, our study shows that *BvUNG* responds to abiotic stress, but more at the level of mRNA amount than the enzymatic activity. BvUNG mRNA was notably induced by both cold and salt stress, albeit with different kinetics. The uracil-removing activity, however, was increased only by cold stress and only in roots, while it was attenuated in roots by salt stress and remained at a steady level in leaves after both types of stress. The situation when mRNA and protein level or activity responses are incongruent is quite common and reflects the regulation of proteins through many other mechanisms, both translational and post-translational [127,128]. Several potential sites of Ser/Thr phosphorylation and Lys acetylation, the modifications known to regulate the activity and protein turnover of human UNG, were predicted in the disordered N-tail and the α_0_ helix of BvUNG. It is thus possible that changes in the total uracil excision activity in response to cold or salt stress result from a complex interplay between *BvUNG* mRNA levels and covalent modifications of the protein, including its parts outside the catalytic domain. The modulation of the uracil-excising activity could itself be adaptive. It is known that both cold and salt stress induce DNA damage and cell death in the cells of various plant species, but apparently through different mechanisms [129,130,131]. While cold stress seems to be genotoxic mostly by reactive oxygen species generation, salt stress is associated with endogenous NO production and protein *S*-nitrosylation [132,133], so cytosine deamination might be increased under these conditions and require tight regulation of UNGs to balance error-free repair and excessive DNA degradation. It remains to be seen what exact physiological roles BvUNG and other plant UNGs play in the response to abiotic stress.

Enhancing DNA repair potential by targeted breeding or genetic engineering has long been viewed as a promising way to crop improvement [134,135,136,137,138]. So far, UNGs have not been studied in such capacity, and the resilience of plants overexpressing these enzymes, or other DNA glycosylases, might be a worthy direction to pursue. On an instrumental side, recent years have witnessed the appearance of genome modification techniques called base editors, which are now widely applied in crops [139,140,141,142]. In particular, cytosine base editors either rely on intracellular UNGs or, conversely, need to inhibit them to regulate the nature of the introduced base changes [143,144]. Understanding the biochemistry and physiology of UNGs in a plant cell will advance our capability to rewrite crop genomes.

## 4. Materials and Methods

### 4.1. Enzymes and Oligonucleotides

*E. coli* uracil–DNA glycosylase was purchased from SibEnzyme (Novosibirsk, Russia). Bacteriophage PBS1 Ugi protein was from New England Biolabs (Ipswich, MA, USA). Oligonucleotides (Table 1) were synthesized at the SB RAS Gene Synthesis Core Facility from commercially available phosphoramidites (Glen Research, Sterling, VA, USA).

### 4.2. Plant Treatment and Extract Preparation

At day 19−21 after planting (three-leaf stage), the plants were separated into three treatment groups (*n* = 3 in each for mRNA extraction, *n* = 9 in each for protein extract preparation). The control group was collected and processed with no further treatment. For cold stress conditions, the plants were incubated at 4 °C for the time indicated (or for 5 h for protein extract preparation). For salt stress, the plants were irrigated with 200 mM NaCl for the time indicated (or 300 mM NaCl for 12 h for protein extract preparation). The whole plants were rinsed in water, sterilized by dipping for 15 s in 0.1% household bleach and 0.05% Tween 20, and washed three times in deionized water. Roots and leaves were separated, dabbed to remove excess moisture, snap-frozen in liquid nitrogen, and stored at −70 °C. The total RNA was extracted by the TRIzol method [145]. The protein extracts were prepared as described [146].

### 4.3. Uracil Excision Assay in Plant Extracts

The reaction mixture (10 µL) included 100 nM oligonucleotide substrate, 20 mM Tris–HCl (pH 8.0), 1 mM DTT, 5 mM EDTA, and various amounts (0.01–20 μg total protein) of the plant extract. EcoUng (0.1 U) was used as a positive control. The reaction was carried out at 37 °C for 1 h and stopped by adding 1 µL of 1 M NaOH and heating for 2 min at 95 °C, followed by neutralization with equimolar HCl. Before loading onto the gel, 6 μL of formamide was added to the sample. The reaction products were resolved by electrophoresis in a 20% polyacrylamide gel/8 M urea, visualized using the Typhoon FLA 9500 imager (GE Healthcare, Chicago, IL, USA) operating in fluorescence mode and quantified with Quantity One v4.6.3 software (Bio-Rad Laboratories, Hercules, CA, USA).

### 4.4. RT-qPCR

Total cDNA was synthesized using a reverse transcription kit (TaKaRa, Kyoto, Japan) according to the manufacturer’s protocol. The specific primers required for qRT-PCR were designed using Primer Premier 5 based on the coding sequence of BvM14UNG. An ABI Prism 7500 PCR system (Thermo Fisher Scientific, Waltham, MA, USA) was used for qRT-PCR with the primers listed in Table 1; the SYBR Green I detection method was employed, and the relative expression level of the gene was calculated by the $2^{-\Delta \Delta C_{t}}$ method [147].

### 4.5. BvUNG Cloning, Mutagenesis, and Purification

Cloning of *BvM14UNG* was described earlier [36]. Briefly, the cDNA was amplified from total leaf RNA of *B. vulgaris* M14 after salt stress using a reverse transcription kit (TaKaRa, Kyoto, Japan) and KOD DNA polymerase (Toyobo, Osaka, Japan). To obtain the expression construct, the full-length open reading frame was subcloned into the pET-15b plasmid at NdeI–XhoI sites to yield the wild-type protein with the N-terminal extension MGSSHHHHHHSSGLVPAGSH-. Site-directed and deletion mutants were produced using the primers listed in Table 1 and Q5 DNA polymerase (New England Biolabs, Ipswich, MA, USA) according to the manufacturer’s protocol. All mutations were verified by Sanger sequencing.

To purify BvUNG NΔ109, *E. coli* BL21(DE3) cells transformed with the pET-15b-BvUNGNΔ109 plasmid were grown at 37 °C with shaking at 250 rpm in 2 l of LB broth containing 100 µg/mL ampicillin to an optical density of A_600_ ~0.6 and induced by 0.2 mM isopropyl β-D-1-thiogalactopyranoside for 3 h at 18 °C. The bacterial pellet was collected by centrifugation at 2000× *g* for 10 min at 4 °C and stored at −80 °C until use. After thawing, the pellet was resuspended in 40 mL of Buffer A (20 mM sodium phosphate (pH 7.5), 500 mM NaCl) supplemented with 1 mM phenylmethylsulfonyl fluoride, and the cells were disrupted by sonication. The lysate was centrifuged at 15,000× *g* for 20 min at 4 °C twice and filtered through Acrodisc 0.45 μm PVDF filters (Pall Corporation, Port Washington, NY, USA). The solution was applied to a 13 mL HiTrap Chelating HP column (GE Healtcare, Chicago, IL, USA) pre-equilibrated with Buffer A, and the column was washed with 65 mL of the same buffer. The protein was eluted with a 20 mL gradient of 25–500 mM imidazole in Buffer A. The fractions were analyzed by SDS-PAGE, and those containing the band of the expected mobility were pooled, diluted with ten volumes of Buffer B (20 mM sodium phosphate (pH 7.5), 1 mM EDTA, 1 mM DTT), and loaded onto a 5 mL HiTrap Heparin HP column (GE Healtcare, Chicago, IL, USA) pre-equilibrated with the same buffer. The column was washed with 25 mL of Buffer B, and the protein was eluted with a 50–1000 mM NaCl gradient in Buffer B (20 mL). The fractions containing the most homogeneous band of the expected mobility were dialyzed against the storage buffer containing 50 mM sodium phosphate (pH 7.5), 400 mM NaCl, 1 mM EDTA, 1 mM DTT, and 50% (*v*/*v*) glycerol and stored at –20 °C. The concentration of the purified protein was determined from the absorption at 280 nm measured on a NanoDrop spectrophotometer (Thermo Fisher Scientific, Waltham, MA, USA).

### 4.6. Steady-State Kinetics

The reaction mixture (10 µL) included 50 mM sodium phosphate (pH 7.5), 100 mM NaCl, 1 mM EDTA, 1 mM DTT, optimized concentration of BvUNG (0.8 nM for the single-stranded U substrate, 1 nM for U:A, 0.2 nM for U:G), and 0.25–17.5 µM DNA substrate. The reaction was carried out for 10 min at 37 °C and stopped by adding 1 µL of 1 M NaOH and heating for 2 min at 95 °C, followed by neutralization with equimolar HCl. Before loading onto the gel, 6 μL of formamide was added to the sample. The reaction products were analyzed by denaturing PAGE as described above. *K*_M_ and *k*_cat_ were determined by fitting the data to the Michaelis–Menten equation using SigmaPlot v11.0 (Grafiti, Palo Alto, CA, USA). All constants were determined from 3–5 independent experiments.

### 4.7. Ugi Inhibition Experiments

The reaction mixtures were as described above but contained 1 nM BvUNG or 2 × 10^−3^ U EcoUng and 0.2 U Ugi when necessary. After 30 min at 37 °C, the reactions were stopped and processed as above.

### 4.8. Screening for UNG Activity in E. coli CJ236 Reporter Strain

Electrocompetent *E. coli* CJ236 cells (FΔ*(HindIII)::cat* (Tra^+^ Pil^+^ Cam^R^)/*ung-1 relA1 dut-1 thi-1 spoT1 mcrA*) were transformed with 50 ng of pET-15b or 60 ng pET-15b-BvUNG or pET-15b-BvUNG H340A (the difference in the mass amount was to equalize the molar amount of the plasmid). The cells were allowed to recover in 1 mL of the SOC medium for 1 h at 37 °C with gentle shaking, diluted 100-fold in SOC, and 50 µL was plated on LB agar containing 100 µg/mL ampicillin. The colonies were counted after the overnight incubation at 37 °C.

### 4.9. Molecular Modeling and Bioinformatic Analysis

Five structure models of BvUNG were generated based on the XP_048492575.1 protein sequence using AlphaFold2 [56], as implemented in the ColabFold v1.5.5 package [148]. An independent model was generated using ESMFold v1.0.3 running on the ESM-2 transformer protein language models [57]. Coarse-grained Monte Carlo modeling was performed in the standalone version of CABS-flex 2.0 [59,60], and 10 runs were performed for each AlphaFold-generated structure. For each run, 50,000 structures were generated, of which 1000 were sampled for analysis and grouped into 10 clusters. The temperature factor of 1.4 was used; amino acid pairs located in helices or sheets more than three positions apart were restrained with a linear potential beyond 3.8–8.0 Å. The obtained trajectories were analyzed in MDTRA [149]. Protein disorder was analyzed with ParSe v2 [61] using the basic classifier alone or that extended to consider π–π, cation–π, and charge effects. PTMGPT2 v0.0.1 was used for post-translational modification site prediction [70].

## Figures and Tables

**Table 1:** Oligonucleotides used in this work ^a^.

ID	Sequence, 5′→3′
Cloning	
BvUNG_fwd	CGTCAAAGAAATGAAATCTCTCCGA
BvUNG_rev	GCTTCAGGCACAAGTCATTCCAT
*RT-qPCR*	
BvUNG_RT_fwd	ATCCTGCCCAAGAATGACGG
BvUNG_RT_rev	CGTGGTTGGGAGCATTACCT
Site-directed and deletion mutagenesis	
BvUNG_H340A_fwd	TAAATCAGCTGCTCCTTCTGGTCTTTC
BvUNG_H340A_rev	AGAATATGGTGCTTAGAC
BvUNG_NΔ109_fwd	ACTGCTGAGCAGAAGTTCAGAATGGAG
BvUNG_NΔ109_rev	ATGGCTGCCGCGCGG
BvUNG_NΔ151_fwd	AAGGAGCTCCTGGTGGAGGATTCGTG
BvUNG_NΔ151_rev	ATGGCTGCCGCGCGGCAC
Enzyme activity and kinetic studies	
23U	Fluo-CTCTCCCTTCUCTCCTTTCCTCT
23ohU	Fluo-CTCTCCCTTCXCTCCTTTCCTCT (X = 5-hydroxyuracil)
23DHU	Fluo-CTCTCCCTTCXCTCCTTTCCTCT (X = 5,6-dihydrouracil)
23hmU	Fluo-CTCTCCCTTCXCTCCTTTCCTCT (X = 5-hydroxymethyluracil)
23Ups	Fluo-CTCTCCCTTCUCTCCTTTCCTpsCpsT
23compA	AGAGGAAAGGAGAGAAGGGAGAG
23compG	AGAGGAAAGGAGGGAAGGGAGAG

^a^ Fluo, 5(6)-carboxyfluorescein; ps, internucleoside phosphorothioate linkage.

**Table 2:** Predicted post-translational modification sites in BvUNG.

Modification Type	Site ^1^
Ser/Thr phosphorylation	*S85*, *S89*, *S91*
*N*-linked glycosylation	**N120**, N203
*O*-linked glycosylation	*S52*, *S53*, *S54*, *T56*, *T64*, *S66*, S357
*S*-nitrosylation	**C133**, C256
Acetylation	**K127**, **K136**, K174, K251
Malonylation	**K127**, K251
Glutarylation	**K121**, **K136**
Glutathionylation	C256
Succinylation	K291, K292
SUMOylation	K337
Formylation	K251, K332

^1^ Sites in the disordered N-tail are shown in italic; sites in the α_0_ helix are shown in bold.

**Table 3:** Kinetic parameters of cleavage of uracil-containing substrates by BvUNG NΔ109.

Substrate	*K*_M_, µM	*k*_cat_, min^−1^	*k*_cat_/*K*_M_, µM^−1^·min^−1^
U	27 ± 12	540 ± 170	20 ± 11
U:A	15 ± 3	230 ± 30	16 ± 4
U:G	3.8 ± 0.8	420 ± 30	110 ± 30

## Data Availability

The original contributions presented in this study are included in the article/Supplementary Material. Further inquiries can be directed to the corresponding author(s).

## Supplementary Materials

The following supporting information can be downloaded at https://www.mdpi.com/article/10.3390/ijms26178221/s1. References [150,151] are cited in the Supplementary Materials.
