# Supplementary material for: Uracil–DNA Glycosylase from Beta vulgaris: Properties and Response to Abiotic Stress
Source: Int J Mol Sci. 2025 Aug 24;26(17):8221. doi: 10.3390/ijms26178221 (PMC12428235; doi:10.3390/ijms26178221)
Supplement: Supplementary file 1 [file ijms-26-08221-s001.zip › Figure_S1.pdf]

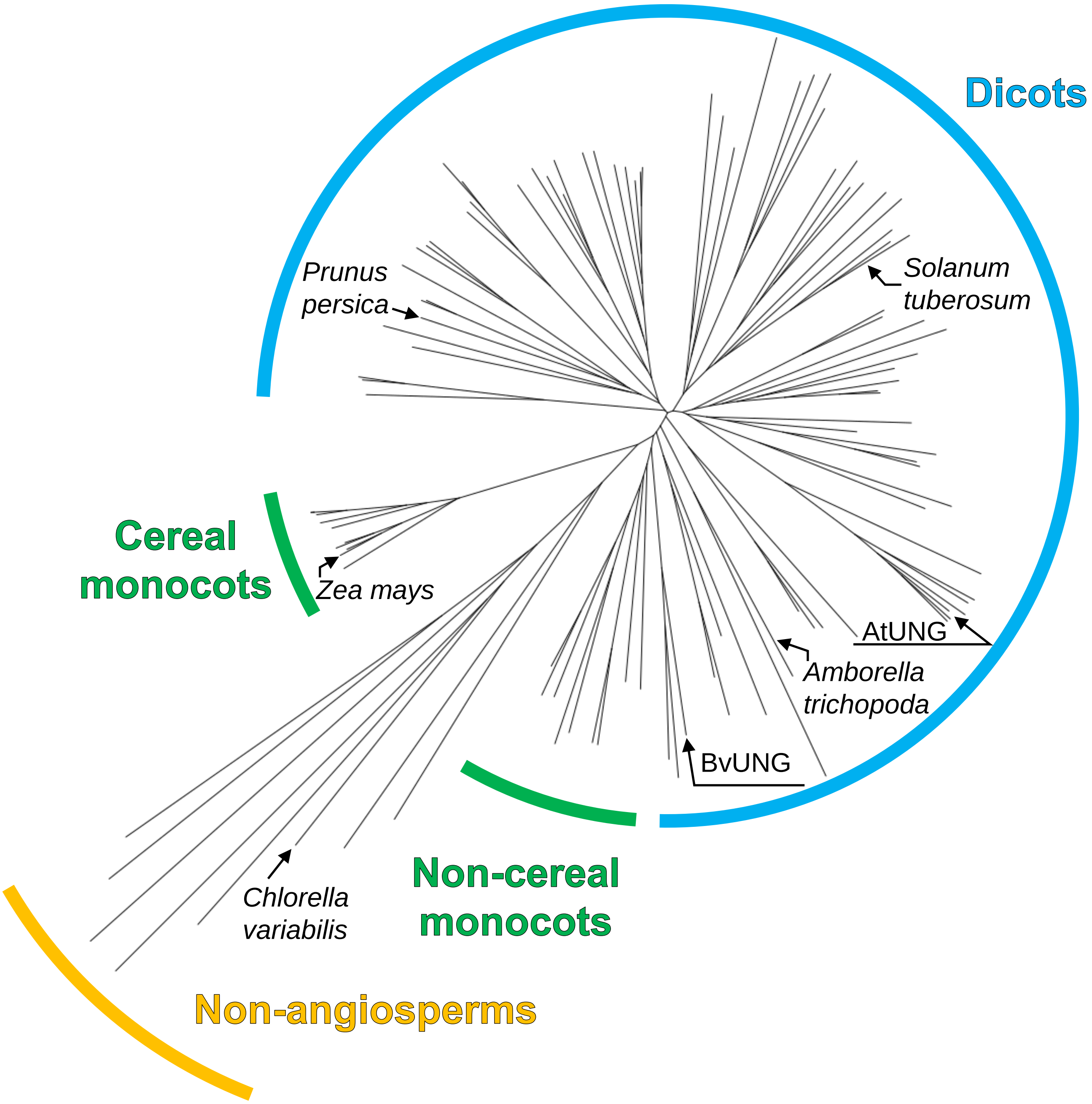

Supplementary Figure S1. Unrooted tree of 108 plant UNG homologs. The homologs were identified through BLAST search limited to Viridiplantae in the RefSeq database [1] using the AtUNG sequence as a query and filtered to keep only the proteins with full-length UDG-F1-like domain, with the taxonomic representation of one sequence per genus. The tree was built based on full-length protein sequences using iTOL [2]. The sequences and the tree in the newick format can be found in Supplementary Files S1 and S2. Arrows mark the positions of BvUNG, AtUNG and the representatives for which the structure models were obtained.
