# Supplementary material for: Uracil–DNA Glycosylase from Beta vulgaris: Properties and Response to Abiotic Stress
Source: Int J Mol Sci. 2025 Aug 24;26(17):8221. doi: 10.3390/ijms26178221 (PMC12428235; doi:10.3390/ijms26178221)
Supplement: Supplementary file 1 [file ijms-26-08221-s001.zip › Figure_S2.pdf]

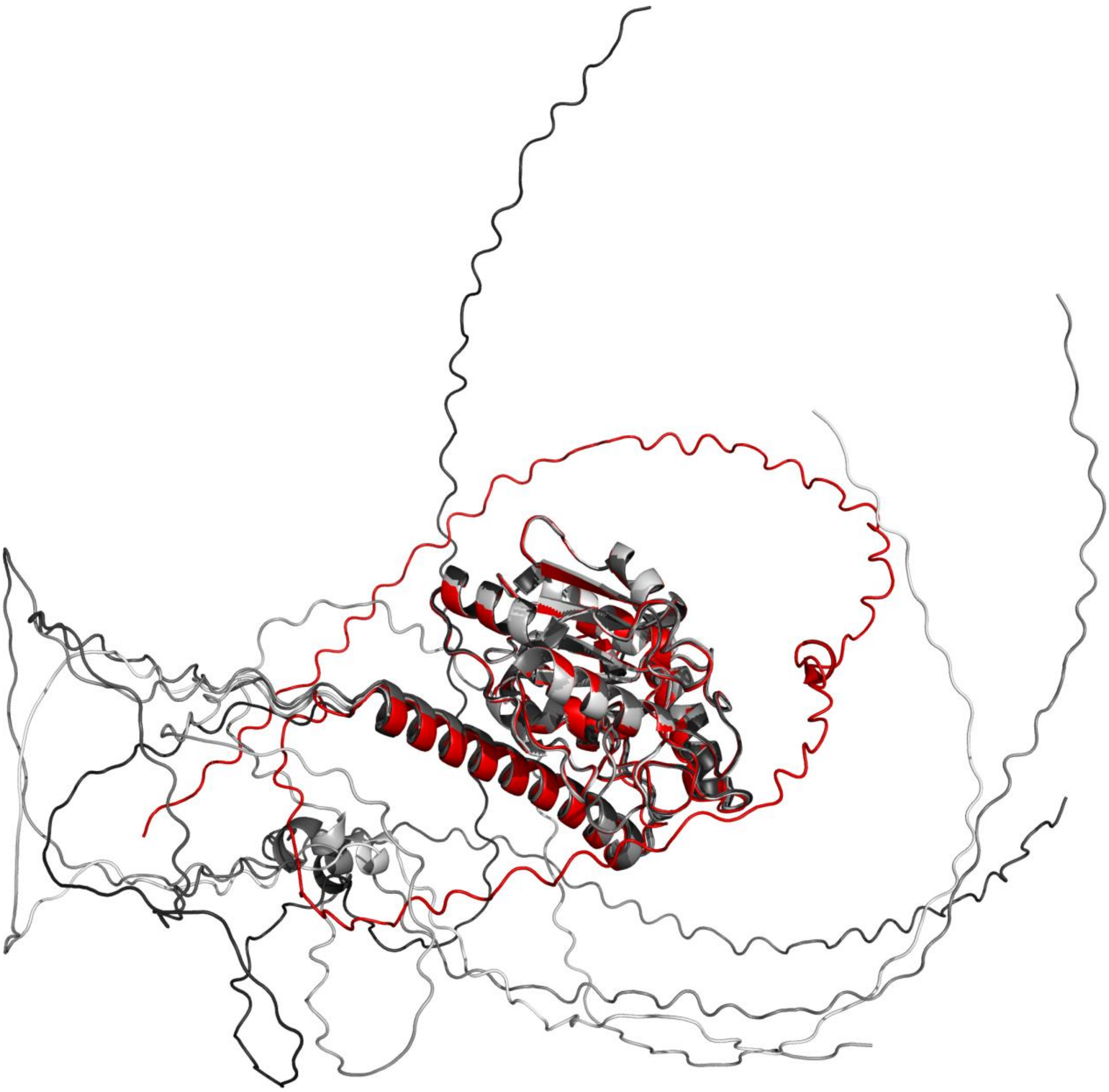

Supplementary Figure S2. BvUNG model generated by ESMFold [3] (red) superimposed over five AlphaFold models (shades of grey).
