# Supplementary material for: Uracil–DNA Glycosylase from Beta vulgaris: Properties and Response to Abiotic Stress
Source: Int J Mol Sci. 2025 Aug 24;26(17):8221. doi: 10.3390/ijms26178221 (PMC12428235; doi:10.3390/ijms26178221)
Supplement: Supplementary file 1 [file ijms-26-08221-s001.zip › File_S3.pdf]

Supplementary References

1. O'Leary N.A., Wright M.W., Brister J.R., Ciufo S., Haddad D., McVeigh R., Rajput B., Robbertse B., Smith-White B., Ako-Adjei D., Astashyn A., Badretdin A., Bao Y., Blinkova O., Brover V., Chetvernin V., Choi J., Cox E., Ermolaeva O., Farrell C.M., Goldfarb T., Gupta T., Haft D., Hatcher E., Hlavina W., Joardar V.S., Kodali V.K., Li W., Maglott D., Masterson P., McGarvey K.M., Murphy M.R., O'Neill K., Pujar S., Rangwala S.H., Rausch D., Riddick L.D., Schoch C., Shkeda A., Storz S.S., Sun H., Thibaud-Nissen F., Tolstoy I., Tully R.E., Vatsan A.R., Wallin C., Webb D., Wu W., Landrum M.J., Kimchi A., Tatusova T., DiCuccio M., Kitts P., Murphy T.D., Pruitt K.D. (2016) Reference sequence (RefSeq) database at NCBI: Current status, taxonomic expansion, and functional annotation. *Nucleic Acids Res.*, **44,** p. D733-D745.
2. Letunic I., Bork P. (2016) Interactive tree of life (iTOL) v3: An online tool for the display and annotation of phylogenetic and other trees. *Nucleic Acids Res.*, **44,** p. W242-W245.
3. Rives A., Meier J., Sercu T., Goyal S., Lin Z., Liu J., Guo D., Ott M., Zitnick L., Ma J., Fergus R. (2021) Biological structure and function emerge from scaling unsupervised learning to 250 million protein sequences. *Proc. Natl Acad. Sci. U.S.A.*, **118:**e2016239118.
4. Xu D., Zhang Y. (2012) *Ab initio* protein structure assembly using continuous structure fragments and optimized knowledge-based force field. *Proteins,* **80,** p. 1715-1735.
